# Endovascular Stenting for Idiopathic Stenosis of the Superior Mesenteric Vein: A Case Report

**DOI:** 10.3390/diagnostics14111187

**Published:** 2024-06-05

**Authors:** Mugur Cristian Grasu, Radu Lucian Dumitru, Gina-Ionela Rusu-Munteanu, Mariana Mihaila, Mircea Manuc, Ioana Gabriela Lupescu

**Affiliations:** 1 Department of Medical Imaging and Interventional Radiology, Faculty of Medicine, Carol Davila University of Medicine and Pharmacy Bucharest, 020021 Bucharest, Romania; ioana.lupescu@umfcd.ro; 2 Department of Radiology and Medical Imaging, Fundeni Clinical Institute, 022328 Bucharest, Romania; gina.m.rusu@icfundeni.ro; 3 Department of Internal Medicine, Fundeni Clinical Institute, 022328 Bucharest, Romania; 4 Department of Gastroenterology, Fundeni Clinical Institute, 022328 Bucharest, Romania; m_manuc@yahoo.com

**Keywords:** superior mesenteric vein, idiopathic superior mesenteric vein stenosis, venous endovascular stenting, long-term outcome

## Abstract

Idiopathic superior mesenteric vein (SMV) stenosis, where no clear causative factor is identifiable, remains a clinical rarity. We present a detailed case report of a patient with idiopathic stenosis of the SMV who underwent successful endovascular stenting. This report outlines the patient’s clinical presentation, diagnostic imaging findings, procedural approach by the interventional radiology team, and subsequent management. Endovascular stenting is a viable therapeutic option for patients with idiopathic SMV stenosis. This case demonstrates that with appropriate interventional and post-procedural management, long-term stent patency and thrombosis prevention can be achieved. The success of this case encourages further investigation into endovascular treatments for venous stenoses.

## 1. Introduction

Superior mesenteric vein (SMV) stenosis is a rare yet clinically significant vascular pathology and can manifest across a spectrum ranging from an asymptomatic state to severe clinical scenarios [1,2].

The etiological landscape of SMV stenosis is multifactorial, straddling congenital anomalies and acquired precipitants. Acquired etiologies often eclipse congenital causes and span post-surgical sequelae, inflammatory processes such as pancreatitis, and extrinsic compression by neoplasms or anomalous anatomical entities [1,2,3]. The diagnosis of this condition is frequently challenging due to its clinical ambiguity, necessitating a judicious use of diagnostic modalities including radiological imaging and, occasionally, explorative laparotomy [1,4].

Clinically, SMV stenosis is insidious, presenting with non-specific gastrointestinal symptoms like abdominal discomfort, emesis, and, in advanced stages, signs indicative of bowel obstruction [5] or gastrointestinal bleeding. The non-specificity often results in diagnostic delays, underscoring the importance of maintaining a high index of suspicion in individuals with relevant clinical histories or surgical antecedents [1].

Imaging is pivotal in the diagnosis of SMV stenosis. Multidetector computed tomography (MDCT) is highly effective in diagnosing this condition. Its rapid image acquisition, superior resolution, and capability to render detailed anatomical visualizations establish it as a fundamental tool in the assessment of complex vascular pathology. MDCT angiography enhances the delineation of vascular structures and facilitates the identification of stenosis, as well as other anomalies such as collateral circulation or concomitant venous thrombosis [6,7,8,9,10].

Idiopathic SMV stenosis, where no clear causative factor is identifiable, remains a clinical rarity, with only one case reported in the literature [11]. The absence of predisposing factors such as a hypercoagulable state [12,13], malignancy [3,14,15,16,17,18], or inflammatory disease [19,20,21] in idiopathic cases necessitates a thorough diagnostic approach to exclude other potential causes.

Therapeutic strategies to SMV stenosis are contingent upon the etiology, symptomatology, and presence of complications [2,3,4,11,14,15,16,17,18,22,23,24,25,26].

The therapeutic approach for symptomatic SMV stenosis can be surgical, including bypass grafting or resection [19,27]. However, with advancements in endovascular techniques, minimally invasive procedures like angioplasty and stenting have emerged as promising alternatives, offering reduced morbidity and shorter recovery times [1,14,15]

The therapeutic regimen is individualized, considering the patient’s overall health status and the specific characteristics of the stenosis [2].

Despite the growing body of literature on SMV stenosis, reports on endovascular management of idiopathic cases are scarce [11]. This case report aims to contribute to the understanding of the role of stenting as a therapeutic option for idiopathic SMV stenosis, addressing a significant gap in the current medical literature.

## 2. Case Report

A 40-year-old male patient was referred to our tertiary center for upper gastrointestinal bleeding, manifested with melena and hematemesis and severe acute posthemorrhagic anemia.

The patient history indicated an urban dweller with no history of tobacco, alcohol, or substance abuse, and no known exposure to toxic substances. The patient had no prior surgical history. Furthermore, there is no significant family medical history to report.

Laboratory tests were in the normal range.

**Figure 1:**
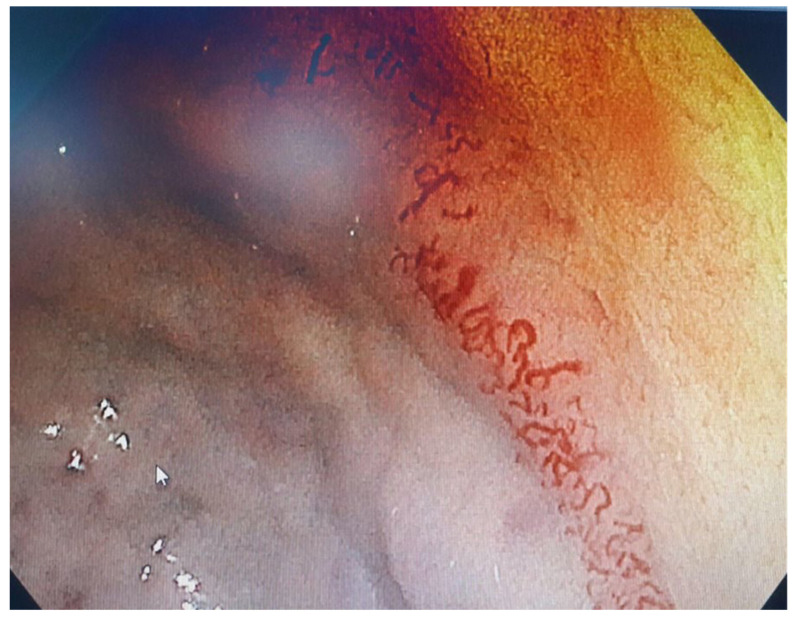
Upper endoscopy—gastric ectatic vascular lesions.

Endoscopic examination identified large ectatic vascular lesions in the gastric region (Figure 1), specifically along the greater curvature and gastric body, extending to the first part of the duodenum, accompanied by a Forrest III classification duodenal ulcer. No evidence of active bleeding was noted, and no therapeutic maneuvers were performed.

**Figure 2:**
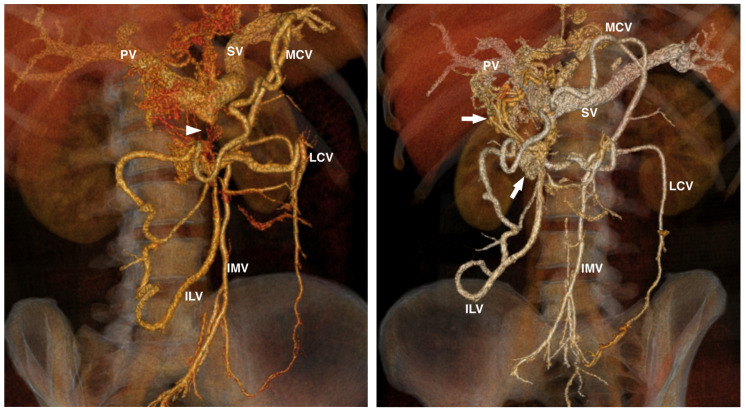
AngioCT (VRT reconstructions)—The distal segment of the superior mesenteric vein exhibits complete stenosis (arrowhead), with mesenteric venous drainage occurring via a network of collateral vessels (arrow) around the pancreatico-duodenal and gastric antral region. Inferior mesenteric vein (IMV), middle colic vein (MCV), left colic vein (LCV), ileo-colic vein (ILV), PV (portal vein), SV (splenic vein).

MDCT angiography revealed a notable stenosis measuring 18 mm in the distal superior mesenteric vein, located 10 mm from the portal confluence. Additionally, there was a proliferation of extensive collateral vessels, predominantly surrounding the stomach (Figure 2).

The case was reviewed by a multidisciplinary team comprising gastroenterologists, surgeons, a diagnostic radiologist, and an interventional radiologist, which reached a consensus to proceed with stenting of the superior mesenteric vein using a transhepatic approach.

Informed consent was appropriately obtained prior to the procedure.

The intervention was executed by the interventional radiology team in collaboration with an intensive care physician and was conducted under deep sedation.

The procedure was performed using a percutaneous transhepatic approach.

A micropuncture introducer set (21G Chiba needle, 0.018-inch guidewire—Neff Percutaneous Access Set—Cook Medical, Bloomington, IN, USA) was used to puncture, under ultrasonographic guidance, an intrahepatic branch of the right portal vein in order to obtain access into the portal system. The 4F introducer sheath was inserted. The 0.018-inch guidewire was exchanged for a 0.035 hydrophilic coating guidewire.

**Figure 3:**
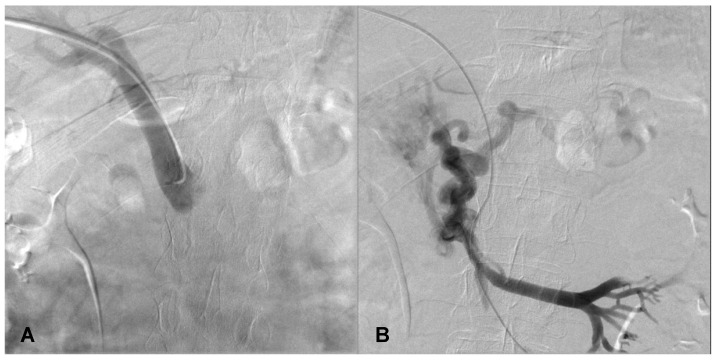
Venography—Portography demonstrating normal hemodynamic flow within the portal vein. (**A**) Post-stenotic injection reveals drainage of the jejunal veins through a collateral network involving the pancreatico-duodenal and antral gastric veins, with no opacification observed in the distal superior mesenteric vein or the portal vein (**B**).

Utilizing a 4F diagnostic catheter (Radifocus™ Optitorque™ Terumo Co., Tokyo, Japan), portography was performed for mapping the portal circulation; however, no abnormalities were detected during the initial phase of the examination (Figure 3A).

A 0.035-inch hydrophilic guidewire was advanced into the superior mesenteric vein and utilized to bypass the stenosis identified in the MDCT scan.

Following successful navigation past the stenosis, the 4F diagnostic catheter was purposefully positioned to facilitate a subsequent angiography.

The venography conducted at the proximal segment of the SMV demonstrated significant stenosis and the presence of multiple collateral venous vessels, predominantly draining around the antral region of the stomach (Figure 3B).

The 4F introducer sheath was exchanged for a 6F sheath.

**Figure 4:**
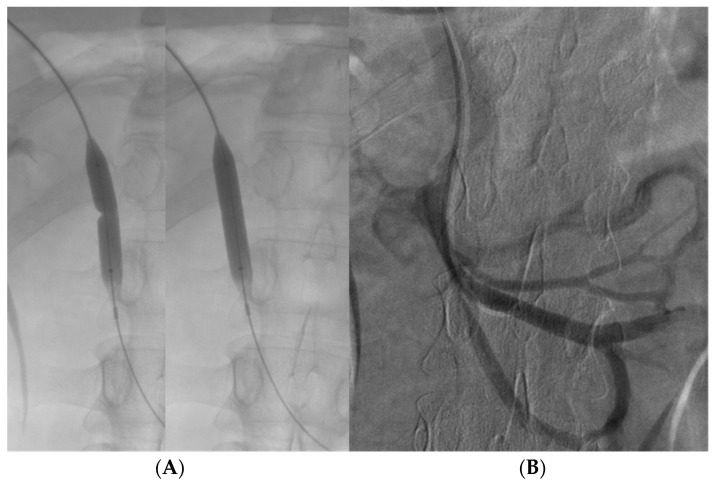
Balloon angioplasty performed on the stenotic segment of SMV using an 8 mm balloon catheter to achieve vessel dilation (**A**). Post-balloon dilation venography reveals a significant reduction in flow through collateral veins, accompanied by the restoration of flow in the distal SMV and the portal vein, indicating successful re-establishment of normal venous circulation in the treated areas (**B**).

The stenosis was dilatated with an 8 mm balloon catheter (Advance^®^ 35LP Low-Profile PTA Balloon Dilatation Catheter—Cook Medical, Bloomington, IN, USA) using a 0.035 Amplatz Extra Stiff guidewire (Extra-Stiff Straight—PTFE-Coated Stainless Steel—Cook Medical, Bloomington, IN, USA) (Figure 4A).

New venography was performed, demonstrating a significant reduction in collateral vessel flow and satisfactory flow into the portal vein (Figure 4B).

Despite achieving a favorable outcome with venoplasty, we opted to stent the SMV stenosis due to the elevated risk of restenosis and to avoid potential complications during future interventions.

From a conservative perspective, stents are generally reserved for cases of intraprocedural recoil or early restenosis following technically successful venoplasty [28]. Funaki and colleagues [29] observed that 12 out of 19 patients undergoing percutaneous intervention for portal vein stenosis post-liver transplantation required stent placement. The patency of the stents was 100% at 3 years. For portomesenteric interventions, the primary stent patency rate was 76% with a mean imaging follow-up period of 8.6 months according to the study by Vamshi K. Mungu et al. [16].

The subsequent intervention involved the placement of an 8 mm self-expanding stent with a 40 mm length (Zilver Flex^®^ Vascular Self-Expanding Stent—Cook Medical, Bloomington, IN, USA) across the identified stenosis.

**Figure 5:**
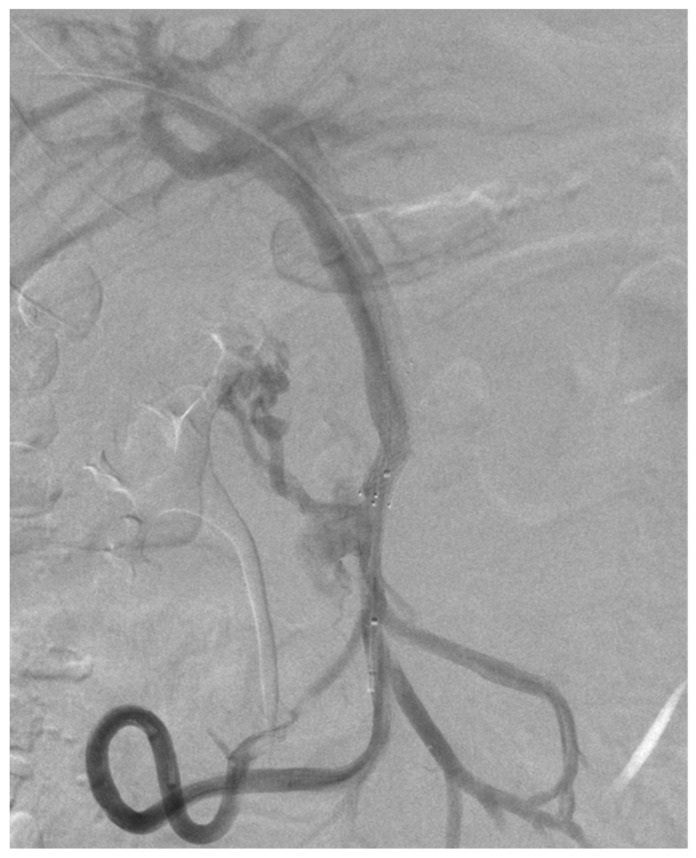
Normal flow in portal vein after stenting stenosis of distal superior mesenteric vein.

The control venography showed an improved flow into the portal vein (Figure 5).

The puncture tract was occluded with a combination of gelfoam and a contrast medium, which was administered through the sheath. While the literature describes various techniques for the occlusion of the puncture tract [30,31], our experience has demonstrated favorable outcomes with gelfoam closure specifically in portal stenting cases involving liver transplant and oncological patients.

There were no complications associated with the procedure.

Five days post-procedure, the patient was administered a therapeutic dose of low-molecular-weight heparin (LMWH).

The patient was discharged five days post-procedure in excellent health status, presenting no complaints.

The anticoagulation treatment prescribed at discharge was apixaban for 8 weeks.

**Figure 6:**
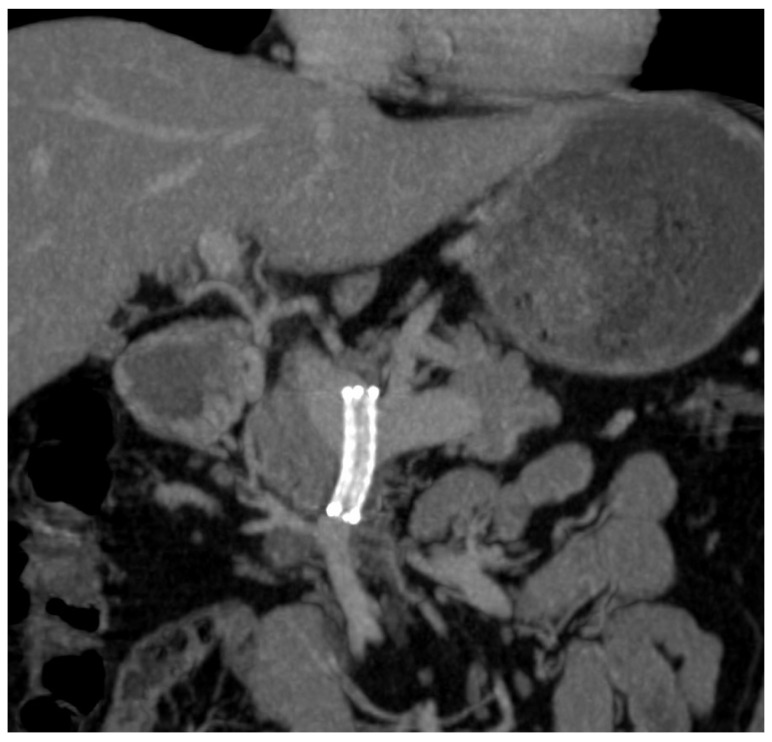
CECT coronal MIP after 1 week—permeable stent in SMV and portal vein.

One week post-procedure, the CT scan confirmed stent patency and demonstrated a significant reduction in collateral vessels (Figure 6).

At one month, the patient underwent follow-up upper endoscopy, which certified the outstanding outcome of the procedure, evidenced by the resolution of the large ectatic veins in the stomach.

**Figure 7:**
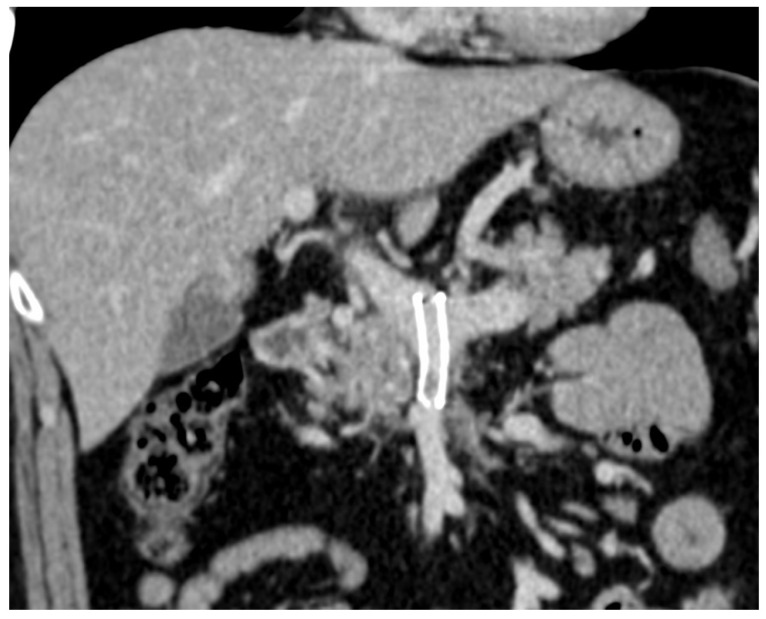
CECT—coronal MIP after 1 year—partially thrombosed stent in SMV.

One year following the procedure, subsequent upper endoscopy revealed the development of novel gastric varices. In accordance with the established follow-up protocol, a subsequent MDCT angiography was performed. The imaging findings indicated partial thrombosis within the stent and venous collateral vessels (Figure 7).

Using the transhepatic approach, in a similar manner as the initial procedure, the venography was performed, and the thrombosis of the stent was confirmed.

**Figure 8:**
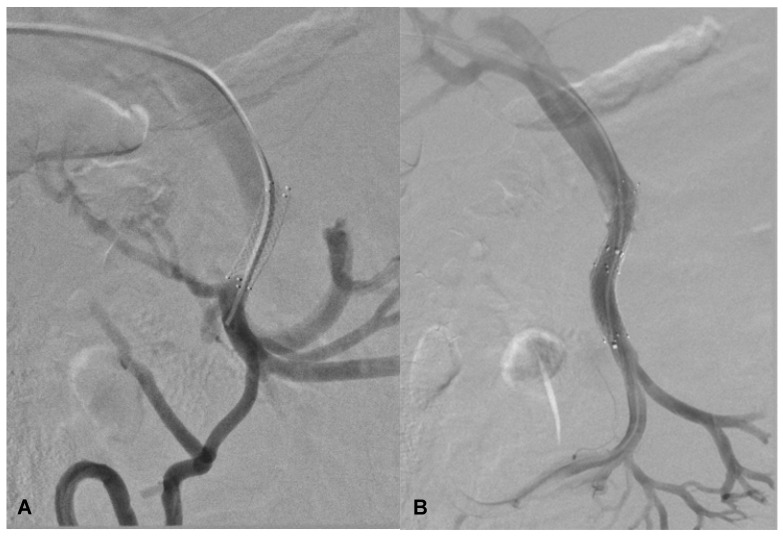
Venography before (**A**) and after (**B**) the balloon dilatation of the previous stent and placement of a second stent in SMV.

The stent underwent dilation, followed by the deployment of a new 8 mm self-expanding stent (Zilver Flex^®^ Vascular Self-Expanding Stent—Cook Medical, Bloomington, IN, USA), ensuring a 7 mm overlap with the pre-existing stent (Figure 8A).

At the end of the procedure, we achieved an optimal flow within the portal vein (Figure 8B).

Three days post-procedure, the patient was administered a therapeutic dose of heparin.

The patient was discharged 3 days post-procedure in excellent health status, presenting no complaints.

The anticoagulation treatment prescribed at discharge, this time, was aspirin and acenocoumarol.

**Figure 9:**
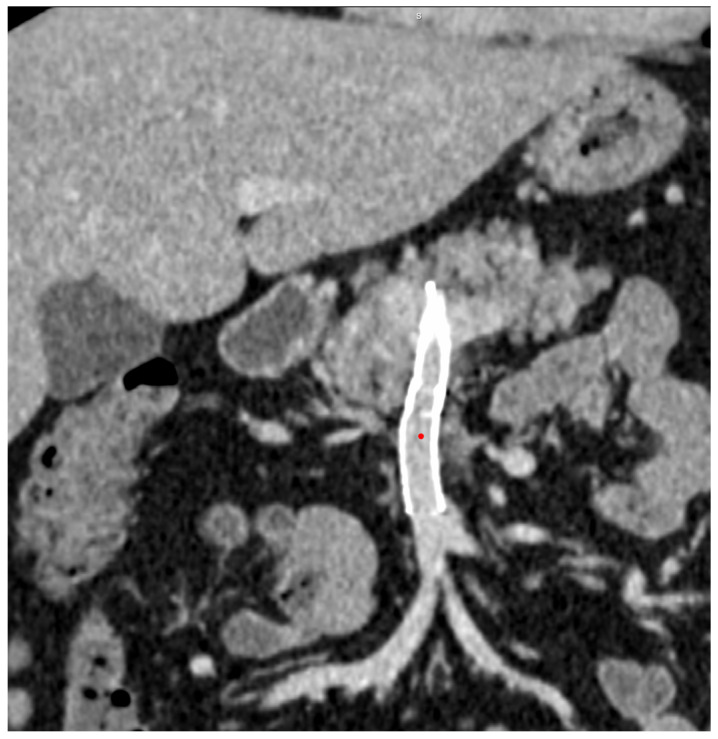
CECT—coronal MIP—5-year patency of the stents placed in the SMV.

Annual surveillance post-procedure, including upper endoscopy, Doppler ultrasound, and CT, was conducted. The stents remained patent over a five-year period with no recurrence of thrombosis (Figure 9).

## 3. Review of the Literature

Over 200 articles have been published in the medical literature over the past decade regarding “superior mesenteric vein occlusion”, yet the vast majority address thrombosis of the superior mesenteric vein.

Among the published articles on mesenteric vein stenosis, most pertain to oncological pathology, inflammatory bowel diseases, or postoperative complications.

Hodgson et al. [19] present three cases of SMV stenosis complicating Crohn’s disease. In all three patients, visceral angiography was conducted, revealing stenosis of the SMV accompanied by the dilatation of draining collateral vessels. The first patient underwent limited right hemicolectomy, which included the resection of 20 cm of the terminal ileum, and was administered propranolol to decrease splanchnic vascular pressure. The second patient received exploratory laparotomy, during which two segments of the small intestine were resected and stricturoplasty was performed. The third patient did not undergo intervention for SMV stenosis; treatment was solely directed towards managing Crohn’s disease.

The endovascular treatment of superior mesenteric vein stenosis is detailed in only a few articles [3,15,23,24,32,33], involving a limited number of patients.

Alden Dimitri and colleagues [18] evaluated the utility and effectiveness of direct percutaneous transhepatic portomesenteric venous stenting (THVS) combined with neoadjuvant chemotherapy to enhance the surgical resectability of locally advanced pancreatic carcinoma. Forty patients with pancreatic carcinoma, demonstrating tumor thrombus involvement of the portal and superior mesenteric veins, were included in the study. These patients underwent THVS followed by neoadjuvant chemotherapy, with a Whipple procedure offered to those who responded favorably. The stented and resected group demonstrated a statistically significant survival benefit (*p* = 0.0422). The authors concluded that THVS used in conjunction with neoadjuvant chemotherapy can improve tumor resectability and overall survival among a selected cohort of patients with locally advanced pancreatic cancer.

Yadav et al. [14] describe a case of bleeding duodenal varices, which were a secondary complication from an occlusion of the SMV due to local infiltration by a pancreatic neuroendocrine tumor. The case was successfully managed with coil embolization of the varices and stenting of the occluded venous segment of SMV after failure of endoscopic glue injection. The authors advocate for the retrograde transhepatic approach for recanalization of the occluded SMV and embolization of associated varices as a viable alternative treatment strategy in similar clinical scenarios.

Beyer and colleagues [2] report a cohort of six patients with superior mesenteric vein stenosis. The objective of the article was to assess the technical and clinical efficacy of percutaneous stenting in the SMV stenosis for symptomatic patients employing self-expanding nitinol stents. The study involved a retrospective analysis of six patients who underwent percutaneous SMV stenting. The etiology of the SMV stenosis included postoperative stricture in three patients, pancreatic carcinoma in one patient, and pancreatitis in two patients. Clinically, three patients experienced symptomatic ascites, two presented with signs of mesenteric ischemia, and one suffered from recurrent gastrointestinal bleeding. The stenting procedure, performed via a percutaneous transhepatic approach using self-expanding nitinol stents, was both technically and clinically successful in all cases, with no peri-interventional complications noted. Stent diameters varied from 6 to 14 mm. Over a mean follow-up period of 6 months (ranging from 2 to 10 months), one patient experienced early stent occlusion two weeks post-placement.

Vamshi K. Mungu et al. [16] evaluated technical success, efficacy, and safety of portomesenteric venous (PMV) intervention for PMV stenosis or occlusion following nontransplant hepatobiliary or pancreatic surgery. The study evaluates a cohort of 42 patients, of which only 5 presented with superior mesenteric vein (SMV) stenosis. The technical success rate was 91%, with a significantly higher success rate observed in patients with stenosis (100%) compared to those with occlusion (56%). The primary presenting symptom was resolved in 28 patients (87%), including all 6 patients (100%) who presented with gastrointestinal bleeding. At a mean imaging follow-up period of 8.6 months (± 8.8 months), the primary stent patency rate was 76%. There was no significant difference in primary stent patency based on the anticoagulation status post-procedure. Two periprocedural complications were reported.

One article [11] documents a unique case of idiopathic stenosis of the superior mesenteric vein (SMV). The case report details an idiopathic SMV stenosis in a patient devoid of any history of pancreatic or hepatic pathologies, or significant abdominal surgical interventions, who developed jejunal varices resulting in chronic gastrointestinal bleeding. This clinical presentation led to melena and anemia. The therapeutic intervention involved percutaneous transhepatic placement of an SMV stent.

## 4. Discussion

The idiopathic etiology of superior mesenteric vein (SMV) stenosis posits a complex interplay of pathophysiological mechanisms.

The idiopathic nature of SMV stenosis in this case raises questions about underlying endothelial dysfunction or subclinical vasculopathic processes [34].

Differential diagnoses such as non-thrombotic mesenteric venous insufficiency and occult neoplastic processes necessitate exclusion through comprehensive diagnostic evaluations, including advanced imaging and hematologic investigations [1,4,11,35].

From a clinical standpoint, this case reinforces the imperative for a high index of suspicion for vascular etiologies in patients with unexplained gastrointestinal symptoms, especially when routine diagnostic modalities yield inconclusive results. The role of interdisciplinary collaboration is underscored, particularly the integration of vascular radiology in diagnostic and therapeutic pathways. Additionally, the post-interventional management, including surveillance strategies like serial Doppler ultrasonography and the potential role of antiplatelet or anticoagulant therapy, remains an area for clinical discretion and further research [2,4,26].

In the realm of endovascular therapy, the choice of the stent type, size, and deployment technique are critical factors. The use of a self-expanding metallic stent in this case was based on its ability to conform to the vessel’s natural anatomy and accommodate potential size variations due to changes in intra-abdominal pressure. The decision against long-term anticoagulation for initial stenting was informed by the idiopathic nature of the stenosis and the absence of hypercoagulability, balancing the risks of stent thrombosis with those of anticoagulant-induced bleeding.

Future research should endeavor to delineate the epidemiology and pathogenesis of idiopathic SMV stenosis more clearly [34].

Prospective studies are needed to compare long-term outcomes between endovascular stenting and traditional surgical approaches [2,4,26].

Longitudinal studies assessing the long-term patency of stents and the incidence of post-stenting syndromes, such as stent migration or intimal hyperplasia, are crucial.

Comparative studies evaluating the outcomes of endovascular stenting versus conservative management with dietary modifications and antispasmodics would further delineate the role of invasive therapy in idiopathic cases.

Investigating genetic or molecular markers that might predispose individuals to idiopathic vascular stenoses could offer insights into targeted therapies or preventative strategies. Additionally, the development of standardized protocols for the management of idiopathic SMV stenosis, including indications for stenting and post-procedural care, would be invaluable in guiding clinical practice [1].

## 5. Conclusions

This case report delineates a personalized approach to the management of idiopathic SMV stenosis, a vascular anomaly that presents both diagnostic and therapeutic challenges. The successful employment of endovascular stenting in this case, resulting in the normalization of venous hemodynamics, underscores its potential as a primary therapeutic modality.

The use of a self-expanding metallic stent, tailored to the anatomical and hemodynamic demands of the SMV, significantly contributed to the successful clinical outcome for the patient. This case reinforces the importance of individualized treatment planning, utilizing advanced imaging modalities and interdisciplinary collaboration for optimal patient care.

However, this case report represents a singular clinical experience, and thus, generalizations of these findings should be approached with caution. The long-term prognosis following stenting for idiopathic SMV stenosis remains an area ripe for investigation. Prospective studies focusing on stent patency, incidence of in-stent restenosis, and potential complications such as stent migration or thrombosis are crucial [2,11,14,26]. Additionally, research into the pathophysiological underpinnings of idiopathic SMV stenosis, including potential microvascular abnormalities or endothelial dysfunction, could yield significant insights into both preventive and therapeutic strategies.

The development of clinical guidelines for the management of idiopathic SMV stenosis, encompassing diagnostic criteria, indications for endovascular intervention, and post-procedural care including anticoagulation therapy, is imperative. Comparative studies evaluating endovascular stenting against conservative management or surgical options could further refine treatment algorithms, enhancing patient outcomes.

In summation, this case report on idiopathic SMV stenosis and its successful management through endovascular stenting contributes valuable insights to the field of vascular medicine. It underscores the need for continued clinical vigilance, advanced diagnostic and therapeutic approaches, and comprehensive research to enhance our understanding and management of this rare vascular condition. The findings advocate for a more nuanced and patient-centric approach in treating mesenteric venous disorders, paving the way for advancements in clinical practice and patient care.

## Data Availability

No new data were created or analyzed in this study. Data sharing is not applicable to this article.

## References

[1] Tilsed J.V.T., Casamassima A., Kurihara H., Mariani D., Martinez I., Pereira J., Ponchietti L., Shamiyeh A., al-Ayoubi F., Barco L.A.B. (2016). ESTES Guidelines: Acute Mesenteric Ischaemia. Eur. J. Trauma. Emerg. Surg..

[2] Beyer L.P., Wohlgemuth W.A., Uller W., Pregler B., Goessmann H., Niessen C., Haimerl M., Stroszczynski C., Müller-Wille R. (2015). Percutaneous Treatment of Symptomatic Superior Mesenteric Vein Stenosis Using Self-Expanding Nitinol Stents. Eur. J. Radiol..

[3] Hellman P., Hessman O., Åkerström G., Stålberg P., Hennings J., Björck M., Eriksson L.G. (2010). Stenting of the Superior Mesenteric Vein in Midgut Carcinoid Disease with Large Mesenteric Masses. World J. Surg..

[4] Chawla Y., Duseja A., Dhiman R.K. (2009). Review Article: The Modern Management of Portal Vein Thrombosis. Aliment. Pharmacol. Ther..

[5] Lin W.C., Chen J.H., Westphalen A.C., Liao C.H., Chen C.H., Chen C.M., Lin C.H. (2016). The Role of CT in Predicting the Need for Surgery in Patients Diagnosed with Mesenteric Phlebosclerosis. Medicine.

[6] Kim H.J., Ko Y.T., Lim J.W., Lee D.H. (2007). Radiologic Anatomy of the Superior Mesenteric Vein and Branching Patterns of the First Jejunal Trunk: Evaluation Using Multi-Detector Row CT Venography. Surg. Radiol. Anat..

[7] Kang H.K., Jeong Y.Y., Choi J.H., Choi S., Chung T.W., Seo J.J., Kim J.K., Yoon W., Park J.G. (2002). Three-Dimensional Multi-Detector Row CT Portal Venography in the Evaluation of Portosystemic Collateral Vessels in Liver Cirrhosis. Radiographics.

[8] Nesgaard J.M., Stimec B.V., Bakka A.O., Edwin B., Ignjatovic D., Oresland T., Frden A.E., Thorsen Y., Andersen S., Negaard A. (2015). Navigating the Mesentery: A Comparative Pre- and per-Operative Visualization of the Vascular Anatomy. Color. Dis..

[9] Sakaguchi T., Suzuki S., Morita Y., Oishi K., Suzuki A., Fukumoto K., Inaba K., Kamiya K., Ota M., Setoguchi T. (2010). Analysis of Anatomic Variants of Mesenteric Veins by 3-Dimensional Portography Using Multidetector-Row Computed Tomography. Am. J. Surg..

[10] Maruyama H., Shiina S. (2021). Collaterals in Portal Hypertension: Anatomy and Clinical Relevance. Quant. Imaging Med. Surg..

[11] Argirò R., Vattermoli L., Di Pietro F., Crociati S., Funari L., Perlangeli V., Floris R. (2022). Percutaneous Transhepatic Stent for Chronic Intestinal Bleeding from Jejunal Varices in Primary Idiophatic Superior Mesenteric Vein Stenosis: A Case Report. Radiol. Case Rep..

[12] Garcia M.C., Ahlenstiel G., Mahajan H., Van Der Poorten D. (2015). Small Bowel Varices Secondary to Chronic Superior Mesenteric Vein Thrombosis in a Patient with Heterozygous Factor V Leiden Mutation: A Case Report. J. Med. Case Rep..

[13] Kujovich J.L. (2011). Factor V Leiden Thrombophilia. Genet. Med..

[14] Yadav A., Gangwani G., Mishra N., Gupta A. (2018). Percutaneous Transhepatic Approach for Recanalization of Superior Mesenteric and Portal Vein in a Patient With Pancreatic Neuroendocrine Tumor Presenting With Bleeding Duodenal Varices: A Brief Case Report. J. Clin. Exp. Hepatol..

[15] Cao G., Ko G.Y., Sung K.B., Yoon H.K., Gwon D.I., Kim J.H. (2013). Treatment of Postoperative Main Portal Vein and Superior Mesenteric Vein Thrombosis with Balloon Angioplasty and/or Stent Placement. Acta Radiol..

[16] Mugu V.K., Thompson S.M., Fleming C.J., Yohanathan L., Truty M.J., Kendrick M.L., Andrews J.C. (2020). Evaluation of Technical Success, Efficacy, and Safety of Portomesenteric Venous Intervention Following Nontransplant Hepatobiliary or Pancreatic Surgery. J. Vasc. Interv. Radiol..

[17] Chua T.C., Wang F., Maher R., Gananadha S., Mittal A., Samra J.S. (2015). Endovascular Stenting of Mesenterico-Portal Vein Stenosis to Reduce Blood Flow through Venous Collaterals Prior to Pancreatoduodenectomy. Langenbecks Arch. Surg..

[18] Alden D., Dudiy Y., Nassiri N., Friedland R.J., Amatulle P., Rosen R.J. (2015). Direct Percutaneous Transhepatic Portomesenteric Venous Stenting in Management of Locally Advanced Pancreatic Cancer. Am. J. Clin. Oncol. Cancer Clin. Trials.

[19] Hodgson R.S., Jackson J.E., Taylor-Robinson S.D., Walters J.R.F. (1999). Superior Mesenteric Vein Stenosis Complicating Crohn’s Disease. Gut.

[20] Ito S., Higashiyama M., Horiuchi K., Mizoguchi A., Soga S., Tanemoto R., Nishii S., Terada H., Wada A., Sugihara N. (2019). Atypical Clinical Presentation of Crohn’s Disease with Superior Mesenteric Vein Obstruction and Protein-Losing Enteropathy. Intern. Med..

[21] Vietti Violi N., Schoepfer A.M., Fournier N., Guiu B., Bize P., Denys A. (2014). Prevalence and Clinical Importance of Mesenteric Venous Thrombosis in the Swiss Inflammatory Bowel Disease Cohort. Am. J. Roentgenol..

[22] Baerlocher M.O., Kennedy S.A., Ward T.J., Nikolic B., Bakal C.W., Lewis C.A., Winick A.B., Niedzwiecki G.A., Haskal Z.J., Matsumoto A.H. (2017). Society of Interventional Radiology: Resource and Environment Recommended Standards for IR. J. Vasc. Interv. Radiol..

[23] Wei B.J., Zhai R.Y., Wang J.F., Dai D.K., Yu P. (2009). Percutaneous Portal Venoplasty and Stenting for Anastomotic Stenosis after Liver Transplantation. World J. Gastroenterol..

[24] Lin C., Wang Z.-Y., Dong L.-B., Wang Z.-W., Li Z.-H., Wang W.-B. (2024). Percutaneous Transhepatic Stenting for Acute Superior Mesenteric Vein Stenosis after Pancreaticoduodenectomy with Portal Vein Reconstruction: A Case Report. World J. Gastrointest. Surg..

[25] Sacks D., McClenny T.E., Cardella J.F., Lewis C.A. (2003). Society of Interventional Radiology Clinical Practice Guidelines. J. Vasc. Interv. Radiol..

[26] Mauri G., Monti L., Pedicini V. (2011). Interventional Management of In-Stent Thrombosis after Superior Mesenteric Vein Stenting. Eur. J. Vasc. Endovasc. Surg..

[27] Shiozaki S., Matsugu Y., Hamaoka M., Ishimoto T. (2022). Superior Mesenteric Vein to the Right Testicular Vein Shunt Operation for Jejunal Varices Bleeding Associated with Extrahepatic Portal Vein Obstruction after Pancreaticoduodenectomy: A Case Report. Surg. Case Rep..

[28] Saad W.E.A. (2012). Portal Interventions in Liver Transplant Recipients. Semin. Interv. Radiol..

[29] Funaki B., Rosenblum J.D., Leef J.A., Zaleski G.X., Farrell T., Lorenz J., Brady L. (2000). Percutaneous Treatment of Portal Venous Stenosis in Children and Adolescents with Segmental Hepatic Transplants: Long-Term Results. Radiology.

[30] Uller W., Müller-Wille R., Grothues D., Schelling J., Zausig N., Loss M., Stroszczynski C., Wohlgemuth W. (2014). Gelfoam for Closure of Large Percutaneous Transhepatic and Transsplenic Puncture Tracts in Pediatric Patients. RöFo -Fortschritte Auf Dem Geb. Der Röntgenstrahlen Und Der Bildgeb. Verfahr..

[31] Dollinger M., Goessmann H., Mueller-Wille R., Wohlgemuth W.A., Stroszczynski C., Heiss P. (2014). Percutaneous Transhepatic and Transsplenic Portal Vein Access: Embolization of the Puncture Tract Using Amplatzer Vascular Plugs Perkutaner Transhepatischer Und Transsplenischer Pfortaderzugang: Embolisation Des Punktionskanales Mittels Amplatzer Vascular Plugs. Rofo.

[32] Agarwal H., Kumar V., Kumar A., Priyadarshini P., Gamanagatti S., Kumar S. (2023). Control of Traumatic Superior Mesenteric Vein Pseudoaneurysm With a Covered Endovascular Stent Using Transhepatic Approach. Am. Surg..

[33] Jeon U.B., Kim C.W., Kim T.U., Choo K.S., Jang J.Y., Nam K.J., Chu C.W., Ryu J.H. (2016). Therapeutic Efficacy and Stent Patency of Transhepatic Portal Vein Stenting after Surgery. World J. Gastroenterol..

[34] Li H., Shu H., Zhang H., Cui M., Gao Y., Tian F. (2022). Idiopathic Myointimal Hyperplasia of the Mesenteric Veins: A Case Report and Scoping Review of Previously Reported Cases From Clinical Features to Treatment. Front. Med..

[35] Akpinar E., Turkbey B., Karcaaltincaba M., Karaosmanoglu D., Akata D. (2008). MDCT of Inferior Mesenteric Vein: Normal Anatomy and Pathology. Clin. Radiol..

